# Real-time *in vivo *imaging of *p16*^*Ink4a *^gene expression: a new approach to study senescence stress signaling in living animals

**DOI:** 10.1186/1747-1028-5-1

**Published:** 2010-01-14

**Authors:** Naoko Ohtani, Kimi Yamakoshi, Akiko Takahashi, Eiji Hara

**Affiliations:** 1 Division of Cancer Biology, Cancer Institute, Japanese Foundation for Cancer Research (JFCR), 3-8-31, Ariake, Koto-ku, Tokyo, 135-8550, Japan

## Abstract

Oncogenic proliferative signals are coupled to a variety of growth inhibitory processes. In cultured primary human fibroblasts, for example, ectopic expression of oncogenic Ras or its downstream mediator initiates cellular senescence, the state of irreversible cell cycle arrest, through up-regulation of cyclin-dependent kinase (CDK) inhibitors, such as p16^INK4a^. To date, much of our current knowledge of how human *p16*^*INK4a *^gene expression is induced by oncogenic stimuli derives from studies undertaken in cultured primary cells. However, since human *p16*^*INK4a *^gene expression is also induced by tissue culture-imposed stress, it remains unclear whether the induction of human *p16*^*INK4a *^gene expression in tissue-cultured cells truly reflects an anti-cancer process or is an artifact of tissue culture-imposed stress. To eliminate any potential problems arising from tissue culture imposed stress, we have recently developed a bioluminescence imaging (BLI) system for non-invasive and real-time analysis of human *p16*^*INK4a *^gene expression in the context of a living animal. Here, we discuss the molecular mechanisms that direct *p16*^*INK4a *^gene expression *in vivo *and its potential for tumor suppression.

## Background

The *INK4a/ARF *gene locus encodes two distinct tumor suppressor proteins, p16^INK4a ^and ARF, whose expression enhances the growth-suppressive functions of the retinoblastoma protein (pRb) and the p53 protein, respectively[1-4]. It has been estimated that more than 70% of established human cancer cell lines lack functional p16^INK4a ^due to promoter methylation, mutation, or homozygous deletion[5-10]. In many instances the deletions affect both p16^INK4a ^and ARF, but a substantial proportion of the missense mutations exclusively affect p16^INK4a^, suggesting that p16^INK4a^, by itself, plays significant and non-redundant roles in tumor suppression[5-10]. Indeed, accumulating evidence suggest that the p16^INK4a ^gene acts as a sensor of oncogenic stress, its expression being up-regulated upon the detection of various potentially oncogenic stimuli, such as cumulative cell division or oncogenic Ras expression, in cultured human primary cells[11-15]. This unique feature of p16^INK4a ^gene expression, together with its ability to induce the irreversible cell cycle arrest termed cellular senescence, raises the possibility that the p16^INK4a ^gene acts as a safe-guard against neoplasia[3,4,16-19]. However since the simple act of placing cells in tissue culture is sufficient to activate *p16*^*INK4a *^gene expression and the levels of *p16*^*INK4a *^gene expression vary depending on the cell culture conditions[20-23], it remains unclear whether the induction of *p16*^*INK4a *^gene expression in cultured human primary cells truly reflects an anti-cancer process or is an artifact of tissue culture-imposed stress.

We believe that *p16*^*INK4a *^knockout mouse is a powerful tool for elucidating the physiological roles of *p16*^*INK4a *^gene expression in vivo[24,25] A limitation of this approach, however, is the developmental or somatic compensation by the remaining *p16*^*INK4a *^family genes (*p15*^*INK4b*^, *p18*^*INK4c *^and *p19*^*INK4d*^) [26-28]. Moreover, the possibility of cross-species differences between human *p16*^*INK4a *^gene expression and mouse *p16*^*INK4a *^gene expression also complicates the interpretation of *p16*^*INK4a *^knockout mouse data[3]. Alternative approaches are therefore needed to supplement the knockout mice studies and to assist in understanding the roles and mechanisms regulating human *p16*^*INK4a *^gene expression *in vivo*.

Bioluminescence imaging (BLI) is an emerging approach that is based on the detection of light emission from cells or tissues[29,30]. Optical imaging by bioluminescence allows a non-invasive and real-time analysis of various biological responses in living animals, such as gene expression, proteolytic processing or protein-protein interactions in living animals [31-36]. Recently, we have generated a new transgenic mouse line (*p16-luc*) expressing the fusion protein of human *p16*^*INK4a *^and firefly luciferase under the control of human *p16^INK4a^* gene regulation[37]. Using this humanized mouse model, we have recently explored the dynamics of human *p16*^*INK4a *^gene expression in many different biological processes in living animals[37]. In this commentary, we will introduce the unique utility of BLI in advancing our understanding of the timing and hence, likely roles and mechanisms regulating *p16*^*INK4a *^gene expression *in vivo*.

### Real-time imaging of *p16*^*INK4a *^gene expression in living animals

**Figure 1:**
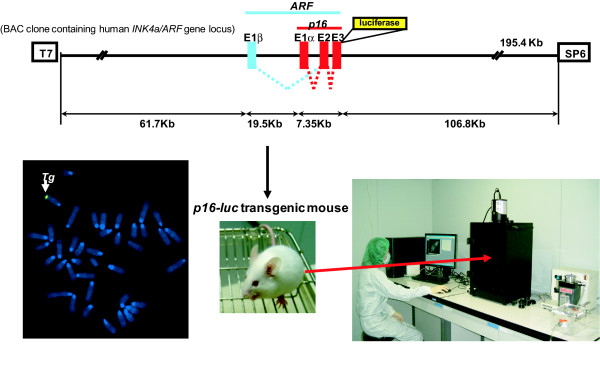
**Strategy for *in vivo *imaging of *p16*^*INK4a *^gene expression**. A large genomic DNA segment (195.4 kb) of human chromosome that contains the entire *INK4a/ARF *gene locus and surrounding sequences was engineered to express luciferase-tagged p16^Ink4a^. FISH technique reveals that the transgenic mice line (p16-luc) contanins a single copy of the human chromosome segment. The arrow shows the transgene. The p16-luc mouse was anesthetized and subjected to *in vivo *bioluminescence imaging after injection of luciferin.

In order to monitor human *p16*^*INK4a *^gene expression as accurately as possible, we used a large genomic DNA segment of the human chromosome that contains the entire *INK4a/ARF *gene locus(Figure 1). Furthermore, this human chromosomal segment was engineered to express a fusion protein of human p16^INK4a ^and firefly luciferase without deleting any genomic DNA sequences of the *INK4a/ARF *gene locus (Figure 1). This is crucial, because BMI-1, which is a negative regulator of *p16*^*INK4a *^gene expression[38], has been shown to bind not only to the promoter region, but also to the intron region of the *p16*^*INK4a *^gene locus[39]. Moreover, the expression of the p16-luc fusion protein enables us to specify *p16*^*INK4a *^gene expression, but not *ARF *gene expression, from this overlapping gene locus.

**Figure 2:**
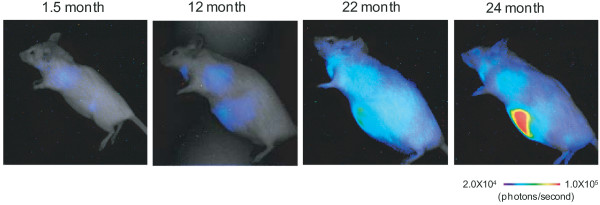
**Real-time bioluminescence imaging of *p16*^*INK4a *^gene expression during aging process *in vivo***. The same p16-luc mice were subjected to noninvasive BLI throughout their entire life span. The level of bioluminescent signals is significantly increased throughout the body during aging.

By monitoring and quantifying the bioluminescent signal repeatedly in the same *p16-luc *mouse throughout its entire lifespan, we were able to unveil the dynamics of human *p16*^*INK4a *^gene expression in the aging process of the transgenic mouse (Figure 2). Importantly moreover, the bioluminescence signal levels correlated well with not only exogenous (human) but also endogenous (mouse) *p16*^*INK4a *^gene expression, indicating that overall regulation of human *p16*^*INK4a *^gene expression is very similar to that of mouse *p16*^*INK4a *^gene expression, at least in mouse cells[37]. This is consistent with the previous notion that the levels of *p16*^*INK4a *^gene expression were increased during the aging process of both rodents and primates [20,40-43]. These results illustrate the potential of the *p16-luc *mice for the analysis of *p16*^*INK4a *^gene expression in response to oncogenic stimuli in vivo.

### The response of *p16*^*INK4a *^gene expression to oncogenic stimuli *in vivo*

Although ectopic expression of oncogenic Ras initiates cellular senescence through up-regulation of p16^INK4a ^expression in cultured normal human fibroblasts[3,4,13,14,44], this is not the case in freshly isolated normal human fibroblasts [23]. It remains, therefore, unclear whether the induction of *p16*^*INK4a *^gene expression by oncogenic Ras expression in cultured cells truly reflects an anti-cancer process or an artifact of tissue culture-imposed stress. To explore this notion in a more physiological setting rather than using the ectopic expression of oncogenic Ras in cultured cells, the *p16-luc *mice were subjected to a conventional chemically-induced skin papilloma protocol with a single dose of DMBA, followed by multiple treatments with TPA. Because this protocol induces benign skin papillomas, more than 90% of which harbor an oncogenic-mutation in the *H-ras *gene[45,46], it appears to be ideal for studying the physiological response to oncogenic mutation in the endogenous *H-ras *gene *in vivo*.

**Figure 3:**
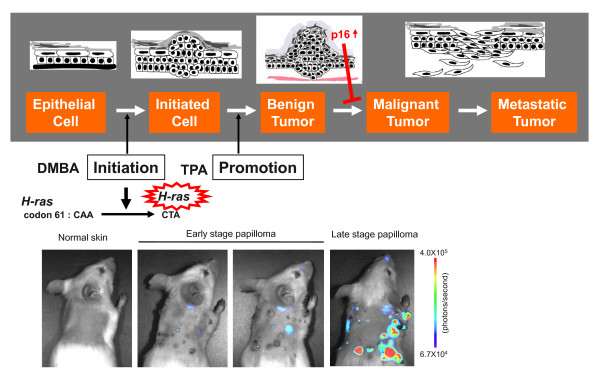
**Real-time imaging of p16^INK4a ^expression during skin papilloma development**. The *p16-luc *mice were subjected to a conventional chemically induced skin papilloma protocol with a single dose of DMBA followed by multiple treatments with TPA. This protocol causes an oncogenic mutation in the *H - ras *gene. Benign skin papillomas began to appear after 7 weeks of DMBA treatment, and continued to grow until 20 weeks or so. However, after that, most papillomas stop growing. So we classified these growing papillomas as the early stage papilloma and non-growing papillomas as the late stage papillomas. The p16-luc mice were subjected to noninvasive BLI, and the significantly elevated bioluminescent signals were detected in the late stage papillomas. The color bar indicates photons with minimum and maximum threshold values.

When p16-luc mice were treated with the DMBA/TPA protocol, benign skin papillomas began to appear after 7 weeks of treatment and continued to grow to a larger size for a further 18 weeks (early-stage papilloma). Although bioluminescent signals were hardly detectable during this time, a significant level of bioluminescent signal was induced as the papillomas stopped growing (late-stage papilloma) (Figure 3). The levels of the bioluminescent signals were well correlated with those of endogenous *p16*^*INK4a *^expression, as well as other senescence markers such as senescence-associated (SA) -galactosidase ( -gal) activity and de-phosphorylation of pRb[37], indicating that the oncogenic Ras signaling derived from the endogenous H-ras gene indeed provokes *p16*^*INK4a *^expression, accompanied by senescence cell cycle arrest, *in vivo*. This also suggests *p16*^*INK4a *^may play important role(s) in late papillomas, presumably preventing the malignant conversion of benign tumors. In agreement with this notion, by 30 weeks after DMBA/TPA treatment, approximately 33% of *p16*^*INK4a *^knock-out mice (C57BL/6 background) had at least one carcinoma, compared with 5% of the wild type mice (unpublished data). These results are also consistent with a previous study showing that the tumor-free survival of DMBA-treated mice was substantially reduced in *p16*^*INK4a *^knockout mice [47].

### Epigenetic regulatory mechanism underlying the *p16*^*INK4a *^gene induction

Given that oncogenic mutation in the *H-ras *gene occurs immediately after DMBA treatment [45], it was puzzling that *p16*^*INK4a *^gene expression was fully induced in the late- but not early- stage papillomas (Figure 3). Interestingly, the levels of DNMT1, which is known to repress *p16*^*INK4a *^gene expression, were significantly increased in early-stage papilloma and subsequently reduced in late-stage papillomas[37]. Intriguingly moreover, the status of the histone 3 Lys 9 methylation (H3K9me), but not the CpG methylation around the *p16*^*INK4a *^gene promoter, was well correlated with the levels of DNMT1 expression during the course of papilloma development[37]. These results, together with a recent observation that DNMT1 possesses an activity to enhance H3K9 methylation through interacting with G9a, a major H3K9 mono- and di- methyltransferase [48], suggest that DNMT1 serves to counterbalance the activation of the *p16*^*INK4a *^gene promoter mediated by oncogenic Ras during skin papilloma development. Of note, the levels of DNMT1 were initially increased by oncogenic Ras expression and subsequently reduced as cells reached the senescence stage in cultured human primary fibroblasts[37]. Together, these results indicate that a similar mechanism is likely to be involved in the regulation of *p16*^*INK4a *^gene expression by oncogenic Ras signaling, both *in vitro *and *in vivo*.

### DNA damage response regulates *p16*^*INK4a *^gene expression through DNMT1

It has previously been shown that oncogenic Ras signaling activates the DNMT1 gene promoter through AP1 [49]. Thus, the induction of DNMT1 expression appears to be caused by a direct effect of oncogenic Ras expression. However, it was unclear how DNMT1 is reduced in the late stage of papilloma development. Our results strongly suggest that the DNA damage response (DDR) triggered by hyper-cell proliferation [50-52] plays critical role(s) in blocking *DNMT1 *gene expression, at least partly, through the elevation of the reactive oxygen species (ROS) level in late-stage papillomas [37]. Since *DNMT1 *gene expression is known to be regulated by E2F [53], and E2F activity is reduced by H_2_O_2_ treatment (unpublished data), it is most likely that ROS regulate *DNMT1 *expression, at least in part, through E2F. These results, together with the observation that depletion of DNMT1 causes up-regulation of *p16*^*INK4a *^gene expression in cultured human cells [54,37], indicate that DDR plays key role(s) in the induction of *p16*^*INK4a *^gene expression through blocking *DNMT1 *expression in the context of Ras-induced senescence *in vivo*.

Because the p53 tumor suppressor is activated immediately after detection of DNA damage, preventing accumulation of DNA damage[55,56], it is possible that p53 might block the DDR pathway activating *p16*^*INK4a *^gene expression. To explore this idea, we again took advantage of using *p16-luc *mice, in conjunction with *p16-luc *mice lacking the *p53 *gene[37]. Indeed, although bioluminescent signals were only slightly induced after treatment with doxorubicin (DXR), a DNA damaging agent, in *p16-luc *mice, this effect was dramatically enhanced by *p53 *deletion, especially in highly proliferating tissues such as the thymus or small intestine[37]. Furthermore, the DDR-pathway activating *p16*^*INK4a *^gene expression and consequent cellular senescence was provoked naturally in the thymus of nearly all mice lacking *p53 *gene at around 10 to 20 weeks after birth[37]. It is therefore possible that *p16*^*INK4a *^may play a back-up tumor suppressor role in case p53 is accidentally inactivated, especially in highly proliferative tissue such as the thymus.

### A regulatory circuit between p53 and p16^INK4a ^tumor suppressors

**Figure 4:**
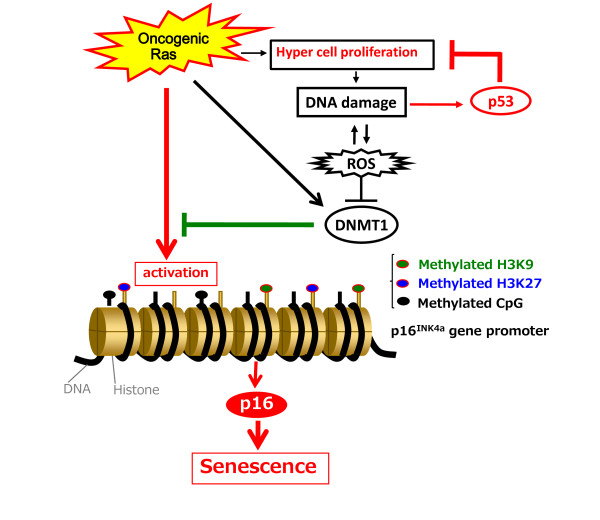
**Cross talk between the p53 and p16 pathways through DDR**. Although oncogenic Ras signaling has a potential to activate *p16*^*Ink4a *^gene expression, this effect is initially counteracted by an elevation of DNMT1 level and thereby causes a strong proliferative burst, resulting in the accumulation of DNA damage. The accumulation of DNA damage activates ROS production, which in turn blocks *DNMT1 *gene expression, thereby causing epigenetic derepression of *p16*^*Ink4a *^gene expression and thus senescence cell cycle arrest. This pathway is counterbalanced by the p53 pathway because p53 is immediately activated by DNA damage and blocks proliferation of damaged cells that cause further accumulation of DNA damage. Thus, the DDR pathway-induced *p16*^*Ink4a *^expression is accelerated in the event of p53 inactivation.

Our results lead to the following model, in which oncogenic Ras signaling has the potential to activate *p16*^*INK4a *^gene expression immediately [13-15], but this effect is initially counteracted by elevation of the DNMT1 levels, which thereby causes hyper-cell proliferation. However, since hyper-cell proliferation tends to cause DNA damage and the elevation of ROS, *DNMT1 *gene expression is eventually reduced by this ROS increase, leading to epigenetic de-repression of *p16*^*INK4a *^gene expression and hence senescence cell cycle arrest (see model in Figure 4). Interestingly, moreover, this pathway is potentiated in the setting of p53 deletion, because p53 tends to prevent the proliferation of damaged cells that would cause a further accumulation of DNA damage (Figure 4) [55,56]. It is therefore most likely that p16^INK4a ^plays a back-up tumor suppressor role if p53 becomes inactivated. In agreement with this notion, it has recently been shown that the levels of *p16*^*INK4a *^gene expression are substantially increased in the mice lacking the *p53 *gene [57]. Moreover, over-expression of Aurora A resulted in a significant induction of p16^INK4a ^expression in the mammary glands of p53 knock-out mice [58]. It is also worth emphasizing that p53 inactivation alone is not sufficient to fully abrogate telomere-directed cellular senescence, but the combined inactivation of p53 and p16^Ink4a ^does do so [59,60]. These results, together with our recent findings[37], help to explain why mice doubly deficient for p53 and p16^INK4a^ exhibited an increased rate of tumor formation [61,62], and why the combination of p53 and p16^INK4a ^loss is frequently observed in human cancer cells [63].

### Concluding remarks

It is, however, clear that all aspects of *p16*^*INK4a *^regulation cannot be explained by the factors described here, and that the *p16*^*INK4a *^gene is subject to multiple levels of control [15,38,39,64-74]. Nonetheless, we have uncovered an unexpected link between p53 and *p16*^*INK4a *^gene expression[37], expanding our understanding of how *p16*^*INK4a *^gene expression is induced by oncogenic stimuli *in vivo*, thus opening up new possibilities for its control. Visualizing the dynamics of *p16*^*INK4a *^gene expression in living animals, therefore, provides a powerful tool for not only helping to resolve issues connecting *in vitro *studies, but also clarifying previously unrecognized functions of this key senescence regulator in various physiological processes *in vivo*.

## Abbreviations used in this paper

CDK: cyclin-dependent kinase; BLI: bioluminescence imaging; DDR: DNA damage response; pRb: retinoblastoma tumor suppressor protein; DNMT1: DNA methyl transferase 1; H3K9: histone 3 Lys 9; H3K9me: histone 3 Lys 9 methylation; ROS: reactive oxygen species

## Ethical approval

The experiments done on mice in figures 1, 2 and 3 followed the guidelines approved by the Committee for the Use and Care of Experimental Animals of the Japanese Foundation for Cancer Research.

## Competing interests

The authors declare that they have no competing interests.

## Authors' contributions

NO wrote the manuscript. KY collected the information required for this commentary article. AT collected the information required for this commentary article. EH wrote the manuscript.
